# Organizational and Functional Status of the Y-linked Genes and Loci in the Infertile Patients Having Normal Spermiogram

**DOI:** 10.1371/journal.pone.0041488

**Published:** 2012-07-23

**Authors:** Anju Kumari, Sandeep Kumar Yadav, Sher Ali

**Affiliations:** Molecular Genetics Laboratory, National Institute of Immunology, Aruna Asaf Ali Marg, New Delhi, India; University of Bonn, Institut of experimental hematology and transfusion medicine, Germany

## Abstract

Male fertility is an orchestrated interplay of loci on the Y chromosome with a number of genes from across the other chromosomes. In this context, micro-deletions in the Y chromosome have been correlated with spermatogenic failure often leading to infertility. However, causes of infertility in the patients with the normal spermiogram have remained unclear and therefore pose another level of challenge. In the present study, we analyzed 64 STSs, studied different Y-linked genes and loci and conducted single nucleotide variant (SNV) analyses in 31 infertile males with normal spermiogram along with 67 normal fertile males (NFMs) to gain an insight into the organization of their Y chromosome. Further, employing quantitative real-time PCR (qPCR), we studied copy number variation of DYZ1 arrays and three genes and mutational status of *SRY* by direct sequence analyses. STS analyses of the *AZFa, b* and *c* regions in these patients showed known and new mutations. Further, copies of *DAZ* and *BPY2* in the patients were found to be affected 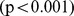 compared to those in NFMs. All the patients had normal copy number of the *SRY* however its sequence analysis (*in silico*) showed mutations in eight patients. In four of these eight patients, *SRY* mutations resulted into truncated proteins. Similarly, DYZ1 analysis showed micro-deletions and it's much reduced copy number 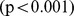 as compared to those in NFMs. Present study in males with unexplained infertility revealed deletions similar to those observed in oligospermic and azoospermic patients. Thus, there are some common but still unknown factors underlying infertility in these patients irrespective of their spermatogenic status. This work is envisaged to augment DNA diagnosis, proving beneficial in the context of *in vitro* fertilization (IVF) and genetic counselling.

## Introduction

Human infertility is a major concern affecting about 15% global population of the reproductive age group [1]. Among the causes of infertility, in half of the cases, male partners are traced to be responsible [2]. A perusal of literature suggests the involvement of the aberrant Y chromosome in the male infertility [3]. The Y-chromosome is male specific, constitutively haploid and inherited by son from the father. The pseudo-autosomal regions (PARs) comprise 3 Mb of the entire Y-chromosome (∼70 Mb) and have homology with the X- chromosome. However, the male specific region of the Y-chromosome (MSY) is devoid of any homology either with the X chromosome or the autosomes thus, escaping meiotic recombination [4]. On account of this, the frequency of mutations and genetic defects in the “intrinsically haploid” genes of the Y-chromosome is quite high as compared to that in X-chromosomal and autosomal genes. Consequently, Y-linked mutations have adverse effects on spermatogenesis and/or normal sperm function [5]. Further, in the absence of recombination of the Y-chromosome, these mutations are faithfully inherited.

Owing to the presence of eight palindromes containing different ampliconic sequences, the MSY reportedly recombine within the region [6], [7] resulting in several large scale Y-chromosomal deletions, detected frequently in the infertile men [8]–[11]. In several studies, falling sperm count and overall deterioration of the semen quality have been reported [12]–[16]. Regardless of the various studies on the male in/fertility, the exact mechanism(s) is still unclear. Apart from the genetic factors, environment and life style are also considered to have a role in male in/fertility [17]–[20]. Among the infertile patients, broadly, there exist two populations; the first in which the male infertility is correlated with decreased sperm count (assessed according to WHO guidelines, 2010 [21]), motility and morphology. The second population comprises males, who despite having a normal spermiogram are infertile. For the second population, analysis at molecular level may highlight the genetic reasons behind their unexplained phenotype. Such studies would reveal the factors which may not be responsible for the spermatogenesis per say, but play a crucial role in the male in/fertility.

The deletions in the euchromatic portion of the Yq have been linked to spermatogenic failures [22]. Three regions, referred to as “Azoospermia factors” *AZFa* (in proximal Yq), *AZFb* and *AZFc* (in the distal Yq euchromatin) have been defined as spermatogenic loci [23]. These *AZF* loci are molecularly screened and mapped for the possible deletions using conserved sequence tagged sites (STSs). Deletions in various *AZF* regions have been correlated with different phenotypes such as *AZFa* region to Sertoli-cell-only syndrome [24]–[26], *AZFb* region to interruption in meiosis-I resulting in spermatogenic arrest [23] and *AZFc* region to hypospermatogenesis leading ultimately to severe oligospermia and azoospermia [27]. Further, *AZFc* and *DAZ* deletions are well documented in the context of spermatogenic failure resulting in infertility [28], [29].

DYZ1 region comprising ∼3.4 kb repeat arrays of about 3000–4300 copies [30] in a normal male constitutes approximately 40% of the Y-chromosome [31]. Each array predominantly contains a pentanucleotide repeat motif 5′TTCCA3′. DYZ1 doesn't recombine; therefore the mutations in the arrays remain unaltered and make it an ideal candidate for studying length and copy number variations. Copy number of this array was found to be drastically affected in cases of repeated abortion, prostate cancer, males exposed to natural background radiation [30] and heavy metals such as groundwater arsenic [32]. Intronless gene *SRY*, located on the short arm of the Y-chromosome encodes a protein, which plays an imperative role in initiating sex determination of the fetus. Mutations in this gene have been linked to various disorders like gonadal dysgenesis and sex reversed cases.

Due to the non-recombining nature of the Y-chromosome, its mutational load may get inherited to the subsequent generations. Therefore, before opting for assisted reproductive technology (ART), the Y-chromosome may be analyzed for micro-deletions, if any. The *AZF* micro-deletions in the patients can be considered as premutations for the complete loss of the Y-chromosome in their sperms thereby increasing the chances to have an XO offspring [33]. These deletions also result in reduction of the normal Y bearing sperms and an increase in nullisomic and disomic (XY) sperms [34]. Such sperms if used for intracytoplasmic injection of the eggs (ISCI) would make the embryos prone to genetic disorders and chromosomal aneuploidies like 45X and 45 XXY. Thus, screening for deletions of the Y-chromosome has prognostic value and implications on subsequent therapeutic options.

Owing to the envisaged correlation between infertility and molecular abnormalities of the Y-chromosome, we studied 31 males with unexplained infertility, having normal spermiograms (according to WHO parameters). Sixty seven NFMs were included as controls. We discerned the status of the Y-linked STSs/loci spanning the *AZF* regions. Further, we assessed status of the *DAZ* genes by SNV analysis and conducted qPCR to ascertain its copy numbers. Molecular analysis of the *SRY* was conducted for its intactness, sequence variation and copy number status by STS mapping, sequencing analyses and qPCR, respectively. Similarly, copy number and mutational status of the DYZ1 arrays were also analyzed in the context of male in/fertility.

## Results

### Sequence Tagged Site (STS) Mapping of *AZF* region

**Figure 1:**
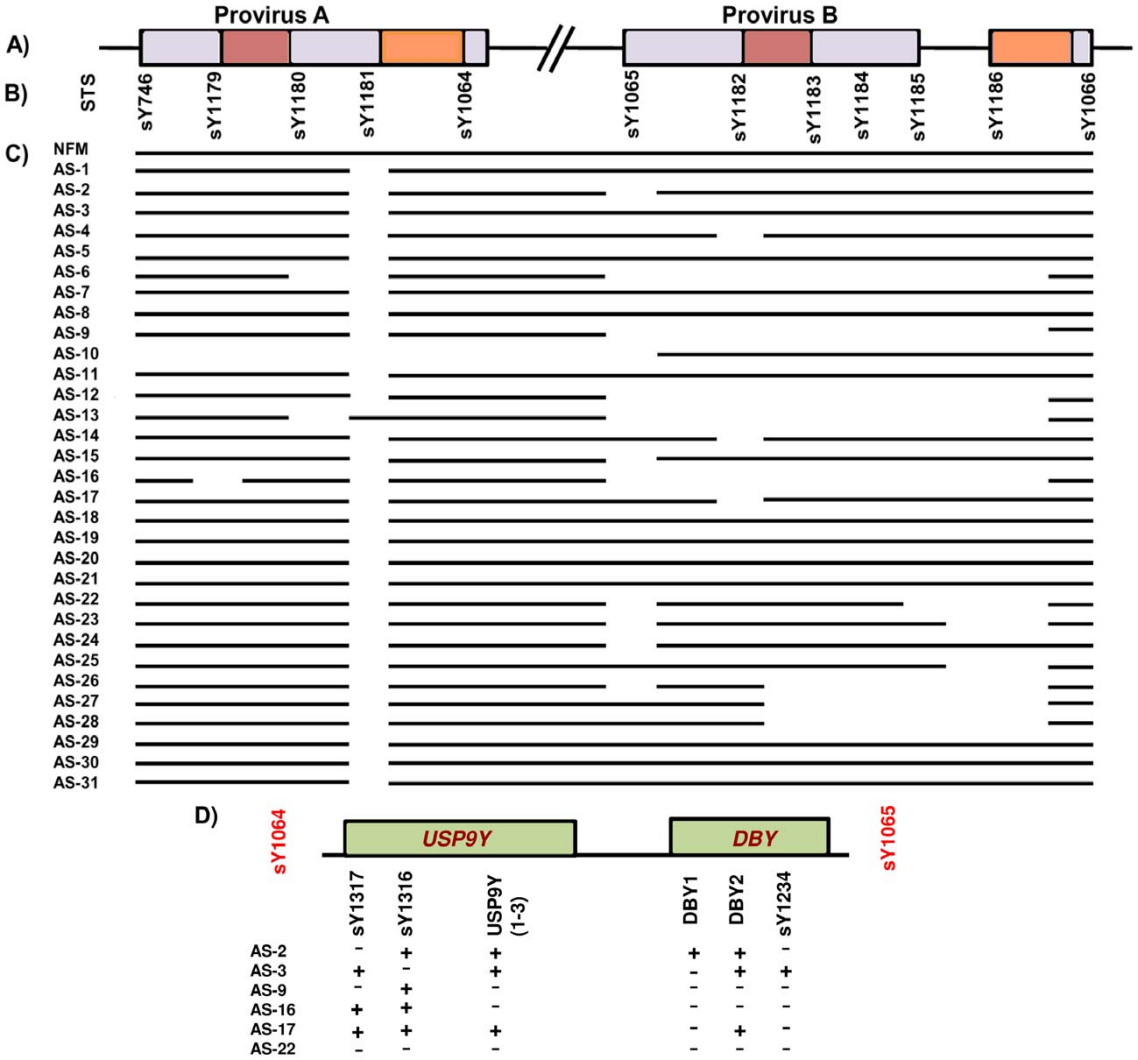
STS mapping of *AZFa* region. (A) Diagrammatic illustration of the *AZFa* showing positions of Provirus A and B regions. The brown and orange bars represent homologous sequences responsible for recombination. (B) STSs employed in mapping of the *AZFa* region. (C) Results of *AZFa* STS mapping using genomic DNA from peripheral blood lymphocytes of the infertile males. NFM signifies the pattern of STS analyses results in normal fertile males. Presence of the corresponding STSs in the patients are indicated by solid line and the gaps denote the absence of the same; patient IDs are shown on the left side. (D) Results of STSs analyses between sY1064 and sY1065. (+) and (−) indicate the presence and absence of the STS analyzed, respectively.

***AZFa*** **: variable deletions were observed:** *AZFa* region was analyzed for HERV mediated recombination using 12 STSs (Table S1). The first HERV recombination event results in deletion of STSs sY1064, sY1065, sY1182, sY1183, sY1184, sY1185 and sY1186. The second recombination event involves deletion of STSs sY1180, sY1181, sY1064, sY1065 and sY1182. The *AZFa* region was found to be intact in all the NFMs. The details of the *AZFa* analyses for patients are shown in Figure 1. Patients AS-6, AS-9, AS-12, AS-13 and AS-16 showed deletion of STSs sY1065, sY1182, sY1183, sY1184, sY1185 and sY1186, a pattern which resembles second HERV mediated deletion except sY1064 that was found to be intact in them. Therefore, to analyze further the deletion breakpoint in these patients, we used STSs between sY1064 and sY1065 including those for genes *USP9Y* and *DBY* (Figure 1D). Five primer pairs across *USP9Y* gene including STSs sY1316 and sY1317 and three primer pairs for *DBY* consisting STS sY1234 were used for all the patients. The patients AS-9, AS-16 and AS-22 showed deletion of *USP9Y*. The sY1316 and sY1317 were found to be deleted in two and three patients, respectively (Figure 1D). *DBY1* STS was found to be deleted in patients AS (3–12, 14–22 and 25) and *DBY2* STS, in patients AS-9, AS-16 and AS-22. Patients AS (2, 9, 16, 17 and 22) were found to have sY1234 deleted, indicating that possible deletion breakpoint in these patients' lies within the *DBY*. The findings of *AZFa* screening in the present study indicate that *USP9Y* and *DBY* genes possibly play a crucial role in the male in/fertility though the mechanism remains unclear. Also, some patients showed random micro-deletions across the *AZFa* region. In addition to this, STSs sY1251 (present at the boundary between Centromere and Yq) and sY1231 (located in *UTY* exon 9) were found to be intact in all the patients, except, AS-9 which showed absence of sY1231 (Table S2). Interestingly, sY1181 was found to be deleted in all the patients except AS-13, but its functional significance remained unclear.

**Figure 2:**
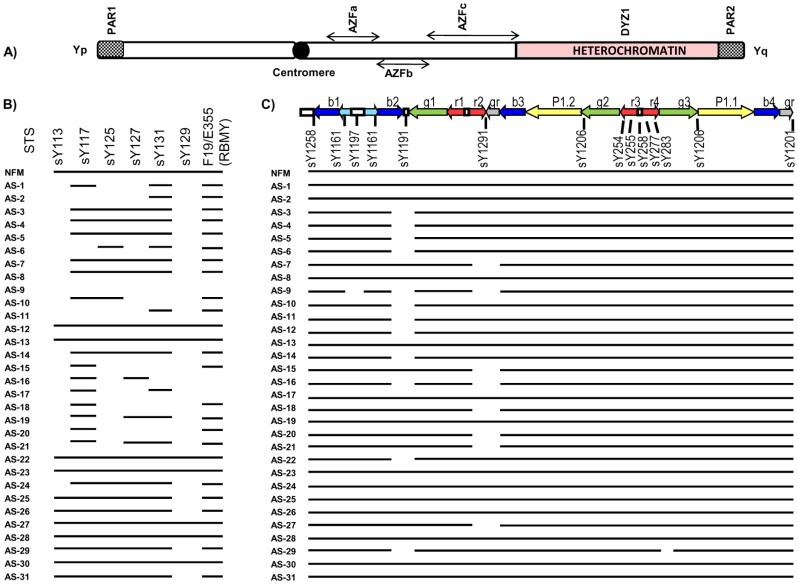
STS mapping of *AZFb* and *c* regions. (A) Diagrammatic illustration of human Y-chromosome. (B) Results of STS analyses of *AZFb* region. STSs analyzed are shown on the top and the patients' ID is shown on the left side. (C) Results of STS analyses of *AZFc* region. The different ampliconic regions present in the *AZFc* region are shown on the top by different colour coding; b1, b2, b3 and b4 are the blue amplicons; g1, g2, g3 and g4 are the green amplicons, likewise r1, r2, r3 and r4 amplicons are in red and gr amplicons in grey. The yellow coloured P1.1 and P1.2 comprise Palindrome 1. The STSs studied for *AZFc* region, are shown below the ampliconic regions. The presence and absence of STSs in the patients are indicated by solid line and gap, respectively.

***AZFb*** **: distinct micro-deletions were seen across the patients:** The *AZFb* region in the patients was analyzed using 7 STSs. The NFMs were positive for all the seven STSs. A diagrammatic illustration of *AZFb* STS screening is shown in Figure 2B. The STSs sY129 and sY113 were found to be deleted in most of the patients. These deletions were confirmed by slot blot hybridization (data not shown). Patients AS-9 and AS-16 showed complete *AZFb* deletion phenotype as reported earlier [35]. *RBMY* gene specific STSs werefound to be intact in all the patients except in AS-9, AS-16 and AS-17.

**Figure 3:**
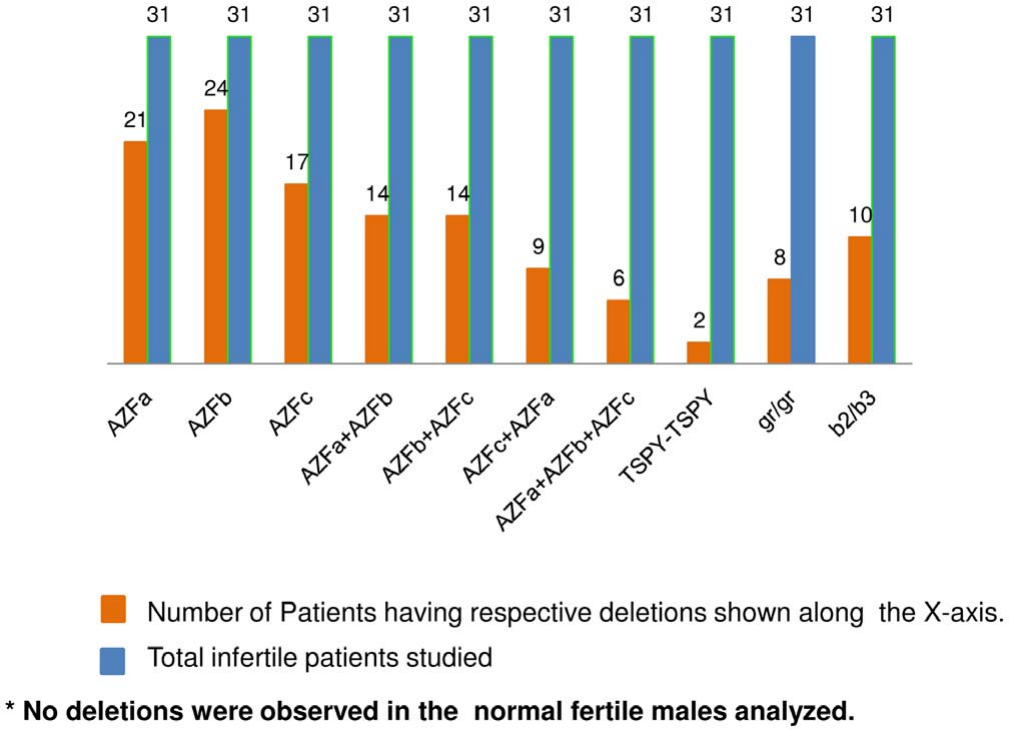
Frequency of the deletions in various *AZF* regions observed in the infertile males. X-axis depicts the deletions observed in different regions of the Y chromosome. The numerical values above the blue and orange bars represent the total number of infertile patients analyzed and number of patients showing the deletions, respectively.

***AZFc*** **: gr/gr and b2/b3 phenotypes were detected in some patients:** The *AZFc* region was analyzed using 20 landmark STSs (Table S1). In NFMs, all the STSs were present. But eight patients AS (7, 9, 15, 16, 18, 20, 21 and 27) showed absence of sY1291 (gr/gr deletion) and twelve patients AS (3–6, 9–12, 14, 16, 22 and 29) showed absence of sY1191 [b2/b3 (g1/g3)] deletion).These gr/gr or b2/b3 (g1/g3) deletion involved loss of two *DAZ* copies and one *BPY2* copy. The sY1197 was found to be deleted in patient AS-9 and sY283 deleted in patient 29 (Figure 2C). The details of deletions in all the patients are shown in Figure 3.

Further, exonic regions of different genes were also studied using specific primers (Table S3). Among these, *CDY1* gene remained unaltered but *CDY2* was affected in 12 patients (Table S2). Also, two patients AS-9 and AS-16 showed deletions of *TTTY17, RPS4Y, XKRY* and *VCY* genes (Table S2).

### SNV analyses showed altered *DAZ* copies

SNV analyses of the *AZFc* region showed partial deletions of *DAZ* gene copies in SNVI, SNVII, SNVIII (sY586), SNVIV, sY587, SNVVI and sY581 (Table 1). The *Fsp1* digested fragments of SNVI and *MboI* digested fragments of SNVII distinguish *DAZ*4 and *DAZ*1, respectively from other *DAZ* copies. Similarly, the *TaqI* digested fragments of SNVIII (sY586) and *AluI* digested fragments of SNVIV discern *DAZ2* from other *DAZ* copies. *DraI* digested fragments of SNVV (sY587) identified *DAZ1/DAZ2* from *DAZ3/DAZ4*, *AflIII* digested fragments distinguished the *DAZ*4 from the rest of the copies and the *Sau3A* digested fragments of sY581 differentiated *DAZ1/DAZ4* from *DAZ2/DAZ3* [11], [28], [36]. The samples, in which the SNV analyses showed different pattern than that of the NFMs (absence of any of the allele) were selected for sequence analyses. The sequencing results of the amplified SNV PCR products in these samples were found to be in accordance with that of the different digested patterns. *DAZ* SNV1 in patients AS (5, 10, 11, 14, 22 and 29) showed presence of allele “A” only. After sequencing, *FspI* site was found to be deleted in these patients due to point mutation (G>A). Similarly, patients AS-20, AS-21 and AS-27 showed presence of allele B only for *DAZ* SNVII; sequencing showed G>A mutation which created an *MboI* site. Presence of allele B of *DAZ* SNVIII (sY586) and SNVIV in patients AS-20, AS-21 and AS-27 was due to T>C point mutation resulting in formation of *TaqI* and *AluI* sites, respectively, both indicating the absence of *DAZ2* copy in these samples. Patients AS (3–6, 10–12, 14, 22 and 29) showed presence of allele A in *DAZ* SNVVI. Sequencing revealed G>T mutation in *AflIII* restriction site responsible for this phenotype. STS Y-DAZ3 was analyzed to ensure presence of the *DAZ3* copy which was found to be deleted in Patients AS (3–6, 10–12, 14, 22 and 29). The sY152 analyzed for *DAZ* (1+4) was present in all the patients except in AS-9 and 16 (Table S2). Thus, SNVs analyses helped to uncover the status of the mutations in the *DAZ* genes and their copy number variation.

**Table 1:** Summary of the results of *AZFc* region based on the SNVs analysis.

S. No.	SNV		Samples having Phenotype		
		A+B	A only	B only	None
**1**	*DAZ*-SNV_I	AS (1–4,6–8,12,13,15,17–21,23–28,30,31)	AS (5,10,11,14,22,29)	-	AS (9,16)
**2**	*DAZ*-SNV_II	AS (1–8,10–15,17–19,22–26,28–31)	-	AS (20,21,27)	AS (9,16)
**3**	*DAZ*-SNV_III (sY586)	AS (1–8,10–15,17–19,22–26,28–31)	-	AS (20,21,27)	AS (9,16)
**4**	*DAZ*-SNV_IV	AS (1–8,10–15,17–19,22–26,28–31)	-	AS (20,21,27)	AS (9,16)
**5**	*DAZ*-SNV_V (sY587)	AS (1,2)	-	AS (3–31)	-
**6**	*DAZ*-SNV_VI	AS (1,2,7,8,13,15–21,23–28,30,31)	AS (3–6,10–12.14,22,29)	-	AS-9
**7**	*DAZ*-SNV_VII (sY581)	All	-	-	-
**8**	*GOLY-*SNV_1	AS (13–19,21–25,27,30,31)	AS (1–12, 20, 26, 28, 29)	-	-
**9**	*BPY2*	AS (1,2,8,13,15,17,19,22–25,28,30,31)	AS (3–7,9–12,14,16,18,20,21,26,27,29)	-	-
**10**	*TTTY4*	All	-	-	-

### Sequencing of *SRY* showed nucleotide variations

**Figure 4:**
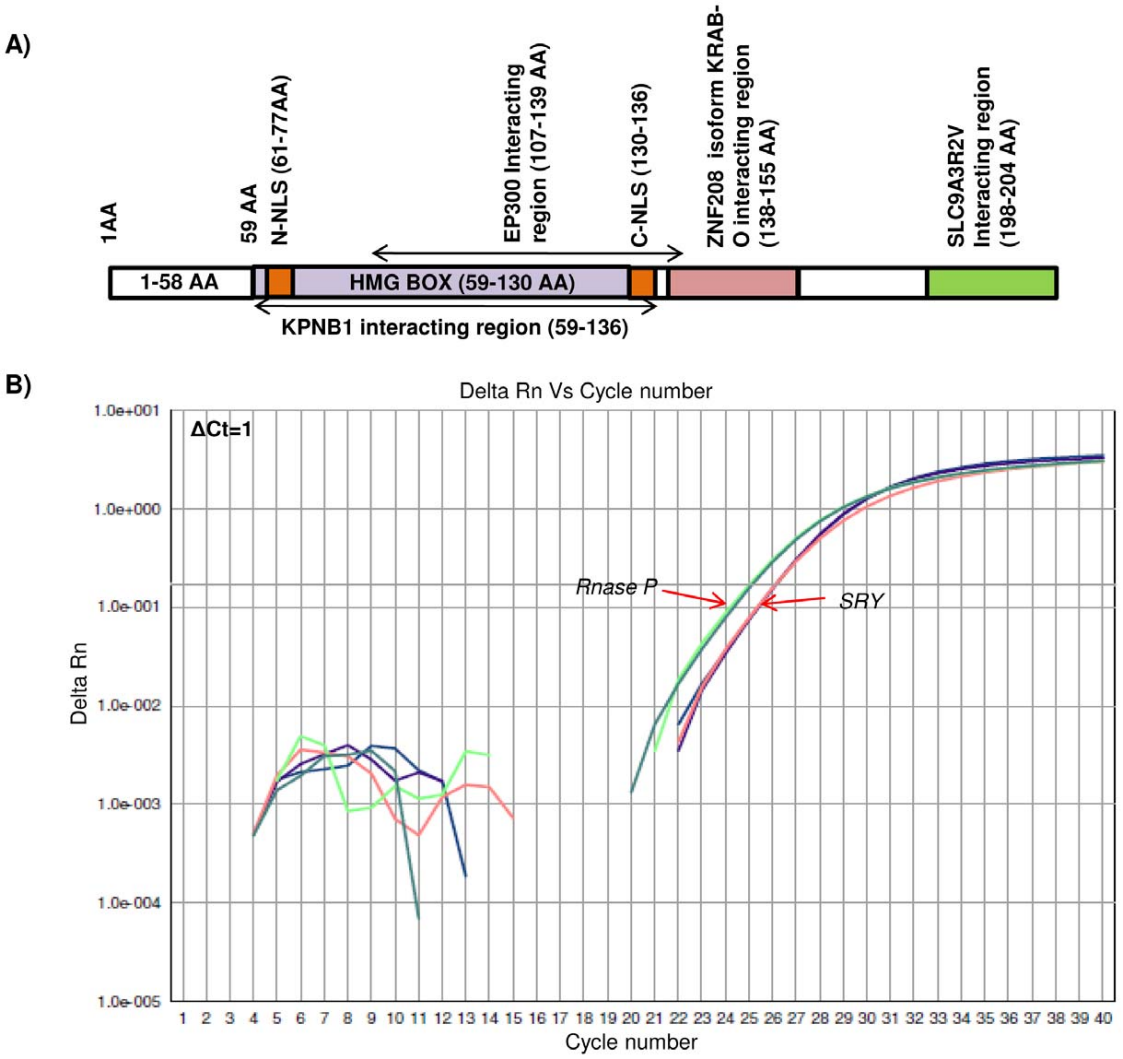
Diagram illustrating positions of important regions and qPCR amplification plot of the *SRY*. (A) Diagrammatic illustration of the functionally important regions of the *SRY* exon, based on the information retrieved from Pubmed. 59–136 AA are sufficient for interaction with *KPNB1*, SOX-TCF_HMG-box present in 59–130AA, contain HMG box and is known to bind minor groove of DNA. 61–77AA are required for nuclear localization and form N terminal nuclear localization signal (N-NLS) of *SRY*. 107–139 AA are sufficient for interaction with EP300, 130–136 AA are required for nuclear localization (C-terminal NLS) and 136 AA is required for acetylation. 138–155AA and 198–204 AA are necessary for interaction with *ZNF208* isoform *KRAB-O* and interaction with *SLC9A3R2V*, respectively. (B) Representative qPCR amplification plot of *SRY* in the infertile patients and normal fertile males. ΔCt = 1 represents one copy of the *SRY*.

All NFMs and patients' samples showed presence of *SRY* gene. The *SRY* sequences of the patients' were submitted to GenBank (Banklt) and their accession numbers are given in Table S4. Multiple alignments of the *SRY* sequence of the representative patients with that of normal male are shown in Figure S1. Patients showing differences at the nucleotide level were analyzed *in silico* to know whether these changes correspond to some alterations in the SRY protein post-translation (using Transeq) and again aligning them to normal SRY peptide. Sequence analyses showed that patients AS (1–8, 10, 12–14, 16, 20–27, 30 and 31) have identical *SRY* CDS compared to that in the normal males. Mutations at the nucleotide level in some patients affect the CDS of the *SRY* gene (Table 2). A diagrammatic illustration of functionally important regions of the *SRY* is shown in Figure 4A. Patients AS-17 and AS-29 showed mutations in the HMG box and *KPNBI* interacting region. In the patient AS-11, mutations were confined to *KPNBI* interacting region whereas in AS-9, the same was observed in the *SLC9A3R2V* interacting region. No such mutations were observed in the *SRY* of the NFMs.

**Table 2:** Details of *SRY* mutations observed in the patients.

S. No.	Samples	Changes in *SRY* gene at nucleotide level	Corresponding changes at protein level (*in silico*)	*SRY* region affected (*in silico*)	Implication of mutation (*in silico* analysis)
**1**	AS (1–8, 10, 12, 13, 14, 16, 20–27, 30, 31)	Identical to normal *SRY*	Identical to normal SRY	None	Identical to normal SRY
**2**	AS-9	350A>C 354_355insC 470delT 478delG	K128X There are large mutations after 117 AA.	*SLC9A3R2V* interacting region	Premature terminated peptide of 127AA.
**3**	AS-11	152A>G 249_250insC 284_285insG 307_308insG 326delT	N51S, many of amino acids after 84 are changed. G181X.	*KPNBI* interacting region	Premature terminated peptide of 180AA.
**4**	AS-15	582A>G	Q194R	-	Q194R
**5**	AS-17	358C>T	H120Y	HMG box and *KPNBI* interacting region	H120Y
**6**	AS-18	28_29insA 166G>A	S10K, V11R, F12I, N13Q, S14Q and D15R, D16X	-	Premature terminated peptide of 15AA.
**7**	AS-19	258G>A	No change in AA	HMG box	Identical protein.
**8**	AS-28	38_39insA 106delT	N13K, S14Q, D15R and D16X	-	Premature terminated peptide of 15AA.
**9**	AS-29	212C>T 357G>C	S71F and M119I	HMG box and *KPNBI* interacting region	S71F M119I

Nomenclature for the description of human sequence variations [66] was followed.

### Deletions and copy number variations were detected in the DYZ1 arrays

**Figure 5:**
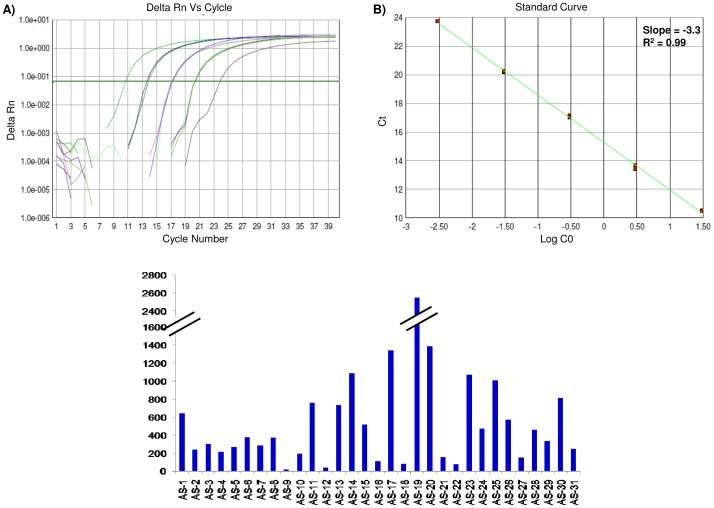
Copy number analysis of the DYZ1 arrays by qPCR. (A) and (B) show representative amplification plot and the standard curves, respectively. (C) represents the copy numbers of DYZ1 arrays across the infertile males.

Primer combinations (PC) used for deletion screening of DYZ1 are explained in the “Methods” section. In all the NFMs, DYZ1 arrays were found to be intact. Amongst the patients studied, AS-9 and AS-16 showed absence of amplicons for PC-1 (3378 bp), 2 (266 bp) and 4 (2095 bp). Patients AS-8 and AS-16 showed deletion of 1564 bp with PC-3. Patient AS-16 showed deletion of 2056 bp and 1612 bp with PC-5 and PC-9, respectively. Patients AS-16 and AS-27 showed deletion of 773 bp with PC-7. Furthermore, the DYZ1 copies were found to vary in the range of 19–2546, which was much below the normal range. The copy number of the NFMs ranged between 2600–4851. The representative amplification plot and standard curve along with the DYZ1 copy number of different patients are given in Figure 5A, B and C. The reduced copy number of DYZ1 array was significantly 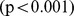 correlated with the infertility status of the patients.

### Copy number status of *SRY*, *DAZ* and *BPY2* genes

**Figure 6:**
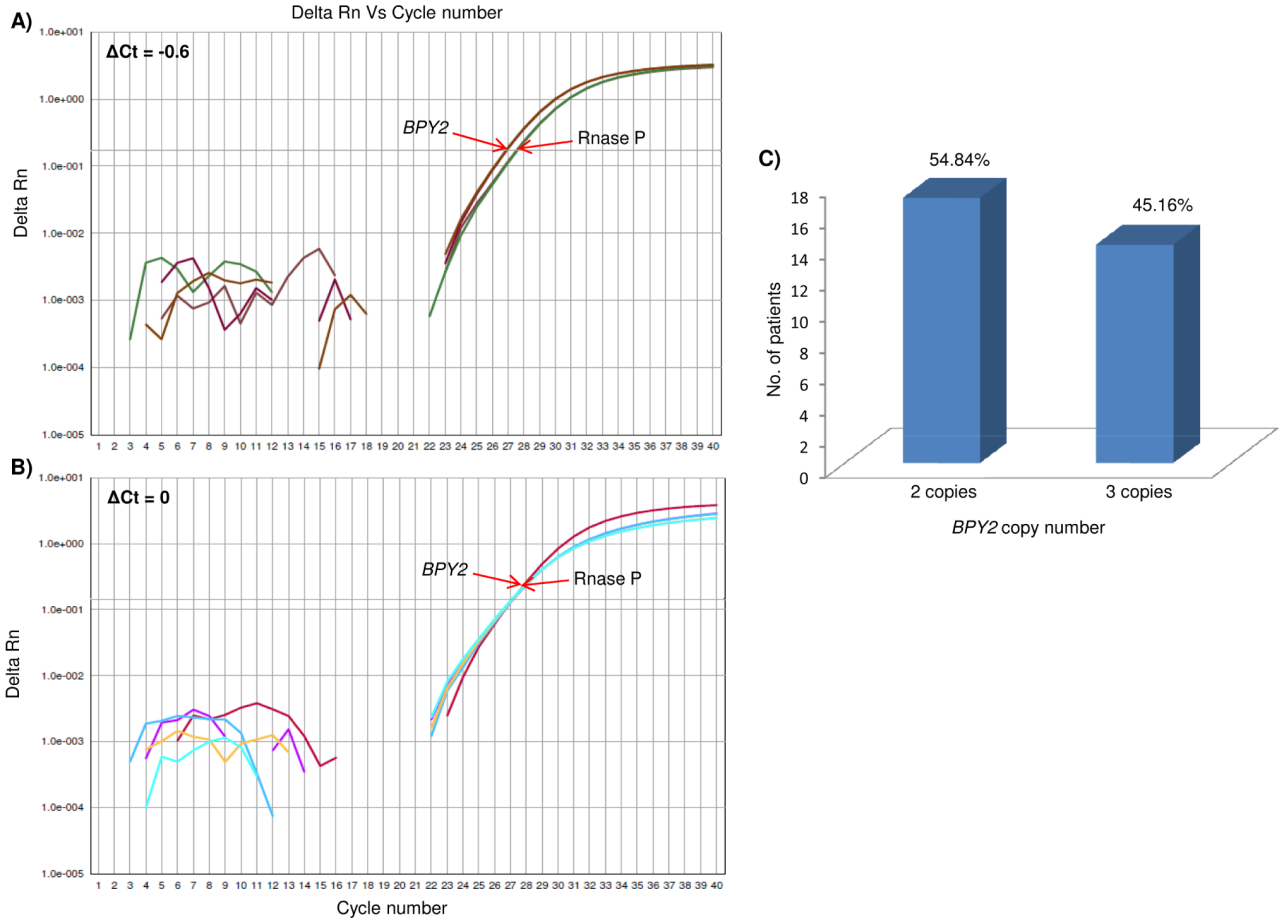
Copy number estimation of *BPY2* gene. (A) and (B) show representative qPCR amplification plots for *BPY2*. ΔCt = 0.6 and ΔCt = 0 correspond to three and two copies of the *BPY2*, respectively (for details on calculations please see “Methods” section). (C) Bar graph shows the frequency of occurrence of 3 and 2 copies across the patients studied.

**Figure 7:**
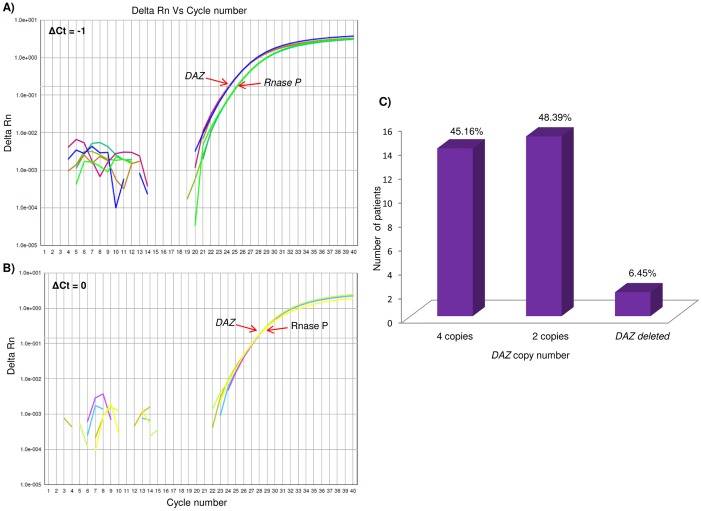
Copy number analysis of *DAZ* gene. (A) and (B) are showing the representative qPCR amplification plots for *DAZ*. ΔCt = −1 and ΔCt = 0 denote four and two copies of *DAZ*, respectively (for details on calculations please see “Methods” section). (C) Bar graph showing distribution of the *DAZ* copies across the infertile patients analyzed.

The representative amplification plots for *SRY*, *BPY2* and *DAZ* copy number calculations are shown in Figure 4B, 6A, 6B and 7A and B, respectively. The copy number of *SRY* across the patients was found to be one, similar to that of NFMs. Copy number of *BPY2* gene in these patients varied from 2–3, while NFMs showed 3 copies. *DAZ* was found to vary between 0–4 copies across the patients. The copy numbers of *DAZ* and *BPY2* genes by TaqMan assays was found to be in accordance with our SNV analysis. The frequencies of the copy number variations in *BPY2* and *DAZ*, across the patients are shown in Figures 6C and 7C, respectively. Table 3 summarizes the details of the copy numbers of these genes in the patients. The patients showed a significant correlation 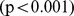 with the reduced copy numbers of *BPY2* and *DAZ* genes.

**Table 3:** Copy number status of *BPY2, DAZ* and *SRY* based on qPCR.

S. No.	Sample no.	*BPY2*	*DAZ*	*SRY*
**1**	AS-1	3	4	1
**2**	AS-2	3	4	1
**3**	AS-3	2	2	1
**4**	AS-4	2	2	1
**5**	AS-5	2	2	1
**6**	AS-6	2	2	1
**7**	AS-7	2	2	1
**8**	AS-8	3	4	1
**9**	AS-9	2	0	1
**10**	AS-10	2	2	1
**11**	AS-11	2	2	1
**12**	AS-12	2	2	1
**13**	AS-13	3	4	1
**14**	AS-14	2	2	1
**15**	AS-15	3	4	1
**16**	AS-16	2	0	1
**17**	AS-17	3	4	1
**18**	AS-18	2	2	1
**19**	AS-19	3	4	1
**20**	AS-20	2	2	1
**21**	AS-21	2	2	1
**22**	AS-22	3	2	1
**23**	AS-23	3	4	1
**24**	AS-24	3	4	1
**25**	AS-25	3	4	1
**26**	AS-26	2	4	1
**27**	AS-27	2	2	1
**28**	AS-28	3	4	1
**29**	AS-29	2	2	1
**30**	AS-30	3	4	1
**31**	AS-31	3	4	1

Copy number of *BPY2, DAZ* and *SRY* genes were found to be normal, i.e. 3, 4 and 1, respectively, in all the normal fertile males.

## Discussion

The human Y-chromosome is male specific and known to harbour genes, important for fertility. Micro-deletions across the Y-chromosome especially *AZF* regions have been argued to be the causes of spermatogenic failure leading to infertility. Deletions in the *AZFa* region are considered to be responsible for the reduced sperm count [9]. However, in the present study, we found micro-deletions across the *AZFa* region of the infertile males having normal sperm count (Figure 1). Deletions of *USP9Y* and *DBY* genes have been associated with spermatogenic failures [37], [38], [39]. Although *USP9Y* and *DBY* deletions alone are not considered to be the major causes of infertility, but construed to be fine tuners of the process of normal spermatogenesis along with other key players [40], [41], [42]. We observed deletions of the *USP9Y* and *DBY* genes in the infertile patients with normal spermiogram. Thus, besides these genes, still other unknown factors are involved with the fertility failure.

Apart from *AZFa* region, we also observed some micro-deletions in the *AZFb* region. Partial *AZFb* deletions have been reported in oligospermic and azoospermic males, suggesting a correlation between reduced sperm count and *AZFb* deletions [35]. Our results indicate that sY113 and sY129 deletions (Figure 2B) in *AZFb* found in most of the patients may be involved along with other key players causing infertility in males with apparently normal spermiogram.

The *AZFc* region is considered as a “hotspot” as most of the infertile cases showed partial deletions (gr/gr, b1/b3, b2/b3 and b3/b4) across this region [10], [34]. Among these, b2/b3 deletions are known to occur in equal frequency in NFMs and infertile patients [43], [44]. Besides these reports, frequency of the b2/b3 deletions was found to be more in the infertile males as compared to that in normal ones [45]. In the present study, none of the normal fertile males showed b2/b3 deletions and the prevalence of b2/b3 deletions in the infertile males was 12/31. Some reports showed profound gr/gr deletion differences between patients and NFMs and considered these deletions significant for male in/fertility [10], [34], [44], [46]–[48]. On the contrary, reports are there that gr/gr deletions are not exclusive to infertile males [43], [45], [49], [50]. However, after thorough reappraisal of the earlier reports, it was recapitulated that these deletions are more common in the infertile males as compared to that in NFMs [51]. In the present study none of the NFMs showed gr/gr deletions but eight patients showed such deletions.

The SNV analyses showed that copies of the *DAZ* and *BPY2* are affected in some patients (17/31), confirming further b2/b3 and gr/gr deletions in these patients. The copy number of the two genes *DAZ* and *BPY2* was found to be normal in 14/31 patients and all the fertile controls. There are disputes about the reciprocity of *DAZ* copy number variations and infertility; on one hand the *DAZ* copies were affected in the infertile males with reduced sperm count [52], [53], conversely the *DAZ1/DAZ2* copies were missing in the infertile males as well as in the normal control samples [50]. Thus, deletions of gr/gr, *DAZ* and *BPY2* may not be directly associated with the spermatogenic failures but play an imperative role in determining the male fertility. One possible explanation for the same can be that these genes are also playing crucial role during post sperm formation.

Co-expression of *CDY1* and *CDY2* was thought to be necessary for the formation of spermatids and spermatozoa [54]. *CDY2* expresses during all the stages of spermatogenesis, while *CDY1* expression remains limited to spermatids and spermatozoa. Therefore, the absence of either of them might affect fertility. In the present study, *CDY2* is found to be affected highlighting its association with infertility.

Mutations in the *SRY* have been reported in sex reversed cases and gonadal dysgenesis [55]–[62]. Though, the copy number of *SRY* in the patients was found to be normal but showed some sequence variations (Table 2). No such mutations were observed in the *SRY* of NFMs implicating possible role of the SRY protein in fertility. Mutations in the patients AS-11, 17 and 29 are in the *KPNB1* interacting region. *KPNB1* (Karyopherin (importin) beta 1) subunit plays important role in nucleo-cytoplasmic transport of the proteins. The import of proteins containing a classical nuclear localization signal (NLS) requires the NLS import receptor, a heterodimer of importin alpha and beta subunits. The importin beta docks the complex at the cytoplasmic side of the nuclear pore complex [63], [64]. The HMG box of *SRY* is essential for binding to and bending of DNA, as well as for transport of the protein into the nucleus [65]. Therefore, any mutation in the importin beta interacting region and HMG box may lead to poor transport of the SRY protein. Also, *SRY* is proposed to switch the foetal development towards maleness by initiating testis differentiation. Owing to this fact, we infer that the mutations observed in the infertile males are likely to be *de novo* somatic variations instead of sporadic changes. Analysis of the samples from the males of the previous generation would have resolved this issue. However, owing to logistic constraints, the same were unavailable for the present study. Thus, besides sex differentiation, *SRY* may have a possible role in determining normal fertility of the males.

Further, we found that the DYZ1 array is also affected in the infertile males. The copy number of DYZ1 array in all the infertile males, with a single exception of AS-19 (DYZ1 copies = 2546) was drastically reduced. Therefore, we hypothesize that the DYZ1 in the heterochromatic region may be necessary for the structural and functional integrities of the Y-chromosome. The heterochromatic region is non-coding and thus considered unnecessary. However, substantial deletions of DYZ1 copies may prove to be detrimental if they include the adjoining functional genes. The reduced copy number of DYZ1 in the infertile males provides direct evidence about its biological significance though the mechanism and consequence(s) of deletion remains unclear.

Male fertility is regulated by a number of genes thus highlighting the complexities of spermatogenesis. In the absence of involvement of a single gene, the accurate DNA diagnosis would remain a challenge. Nonetheless, detailed information is envisaged to be useful to assess the structural and functional status of the Y-chromosome in the context of IVF, DNA diagnostics and ART. Assessment of Y micro-deletions in the infertile patients is particularly relevant for genetic counselling of the infertile couples.

This work demonstrates that infertility in the human males having normal spermiogram is caused due to aberrations of the several Y chromosome linked genes and loci including DYZ1 region. Thus, semen samples used for IVF and ART may be checked for all the possible candidate genes and loci for their normal status. In addition, follow up information may be made available to fine tune the genotype-phenotype correlation in the context of in/fertility.

## Methods

### Ethics Statement

Present study was approved both, by the Institutional Human Ethical Committee of the National Institute of Immunology, New Delhi and the Bio-Ethical Committee, Aligarh Muslim University, Aligarh. Peripheral blood lymphocytes (PBLs) and semen samples were collected from the patients as a diagnostic practice with their informed consent. Most of the patients gave their oral consents as being illiterate villagers. Owing to this, their verbal consents were faithfully recorded. Subsequently, the same was duly reported to both the committees and final approval to conduct the present study was obtained.

### Subjects

Of all the couples reporting at the Department of Endocrinology, J.N. Medical College, Aligarh Muslim University (AMU), Aligarh, thirty one infertile males (median age 32 years) were included in the present study. These couples were not able to conceive following twelve months or more of unprotected intercourse. Both the partners were examined for routine tests. The female partners were found to be normal with respect to their ovulation, hormonal tests (FSH, estrogen and progesterone), tubal and uterine assessment. For the male partners routine semen analyses for sperm count, motility and viability were performed in accordance with WHO (2010) criteria. All the patients were found to be normal with respect to the above parameters. Thus, these patients suffered primary infertility with unexplained causes. The details of the patients with respect to their semen parameters are given in Table S5. The persons having any chromosomal abnormalities (PBL karyotyping), congenital absence of vas deferens, known infertility caused by cystic fibrosis, testicular tumours or undergoing chemotherapy and radiotherapy were excluded from the present study. PBLs in excess of the diagnostic requirements were processed for genomic DNA isolation. Sixty seven NFMs were also included in the present study as controls. All the NFMs had normal spermiogram that were found to be in accordance with the WHO criteria [21].

### Sequence Tagged Site (STS) Mapping of the *AZF* region

*AZF* region of the Y-chromosome was analysed for micro-deletions using STS end point PCR amplification. A total of 64 STSs were selected from the MSY Breakpoint mapper for screening the *AZFa, b, c* regions in the patients and control samples (Table S1). PCR conditions for the same can be retrieved from the NCBI (http://www.ncbi.nlm.nih.gov/pubmed/). The end point PCR amplifications were performed in 25 µl reaction volumes containing 5X Go Taq reaction buffer (Promega Madison, USA), 200 µM dNTPs (Bio Basic Inc. Toronto, Canada), 1 IU Go Taq polymerase (Promega, Madison, USA) and 100 ng of genomic DNA. Primers for β-actin and sY14 (Table S3) were used for ascertaining the quality of genomic DNA samples. Genomic DNA samples from PBLs of NFMs were used as positive controls for STS mapping. Genomic DNA from female and a reaction without template were included as negative controls for each STS. The amplified products were resolved on agarose gels of appropriate concentrations (depending upon the size of the amplicons) and stained with ethidium bromide to view under UV. Each STS reaction was carried out thrice before confirming the same to be negative.

### Single Nucleotide Variants (SNV) analyses

The *AZFc* region was further analyzed for 7 SNVs in the *DAZ* region [28], [36] and one SNV each for *GOLY1*, *BPY2* and *TTTY4* (Table 1) by PCR-restriction fragment length polymorphism. Besides, three landmark STSs DAZ-RRM3 (for *DAZ1* and *DAZ4*), Y-DAZ3 (for *DAZ3*) and *DAZ* 1+4 (sY152) were screened in the *DAZ* region. The primer sequences, accession numbers, product sizes and restriction enzymes used for SNV analyses are given in Table S6. Standard PCR conditions were followed for the analysis of the SNVs and each SNV reaction was performed in a 50 µl reaction volume. An aliquot of 10 µl from each amplified PCR products was analyzed on agarose gel for the presence of respective fragment. Upon confirmation, the remaining PCR product was precipitated by addition of 5 µl of 3 M sodium acetate and 150 µl of absolute ethanol and incubated at −70°C for 3 hours. Subsequently, this was pelleted down at 13,000 rpm for 20 minutes at 4°C, washed in 70% ethanol and dissolved in 10 µl water. The purified PCR amplicons were further subjected to restriction digestion using appropriate restriction enzyme and buffer. The digests were resolved on 3% agarose gel, stained with ethidium bromide and visualized under UV.

### PCR amplification, Cloning and Sequencing of *SRY*

Three sets of primers *SRY*1, *SRY*2 and sY14 (see details in Table S3) were used to study *SRY*. Primer pair *SRY*1, encompassing HMG box region of the human *SRY* was used to amplify genomic DNA from the different patients and products were resolved on agarose gel. The resolved amplicons were then sliced and eluted using gel extraction kit (Qiagen, Hilden, Germany). Eluted DNA was cloned in pGEM®-T easy vector (Promega, Madison, USA). Five recombinant clones each, from all the patients and NFMs were sequenced on Applied Biosystems 3130xl genetic analyzer using ABI PRISM®Big Dye®terminator v 3.1 cycle sequencing kits (Life technologies, California, USA). PCR conditions were set as 96°C for 1 minute, followed by 25 cycles each consisting of 96°C for 10 seconds, 50°C for 5 seconds, and 60°C for 4 minutes. After cycle sequencing, extension products were purified to remove any unincorporated dye-labelled terminators using ethanol-sodium acetate precipitation method followed by washing in 70% ethanol. Hi-Di™ Formamide (Life technologies, California, USA) was added, samples were heat denatured, chilled on ice and loaded on ABI 3130xl genetic analyzer. The sequences were analyzed using Run 3130xl Data Collection Software v3.0, Sequencing Analysis 5.3.1 and Generunner. The *SRY* sequences of the patients and NFMs were aligned to the reference sequence in GenBank (NM_003140.1) using online tool ClustalW.

### Analysis of DYZ1

DYZ1 arrays in different patients were screened by end point PCR for their intactness. Details of primers and their combinations are listed in the tables S3 and S7, respectively. The copy numbers of the DYZ1 arrays of the patients were calculated by qPCR based on absolute quantification method using genomic DNA as template and SYBR green dye (Life technologies, California, USA). The qPCR reactions were performed on Sequence Detection System-7500 (Life technologies, California, USA). Ten fold dilutions of cloned DYZ1 plasmid (∼3.4 Kb *Hae*III digest) were used for generating standard curve starting with 20 crore (2×10^8^) copies. All the reactions were carried out in triplicates using three different concentrations of the template DNA of each patient along with the control samples. The standard curves had a slope of −3.3 to −3.5 and R^2^ value of >0.99. Copies of the DYZ1 arrays were calculated by extrapolation of the standard curve obtained with known copies of the recombinant plasmid.

### Copy number calculation of *SRY*, *DAZ* and *BPY2* genes

Copy number of the *SRY*, *DAZ* and *BPY2* were calculated using TaqMan probes for the respective genes. *SRY* (assay Id. Hs00243216_s1), *DAZ* (assay Id. Hs00414014_m1) and *BPY2* (SARBPYF (forward primer) TGGAGTCTGCCAAAACAAGGG, SARBPYR (reverse primer) CAGAGCAGGAGAGTCTCATCAC and SARBPYP (Probe) 6FAM-CACATATTGCGGAGTCCAGCACCCAGG-MGB) assays were procured from ABI, USA. All the reactions were performed in triplicates on Sequence Detection System, 7500 (Life technologies, California, USA). The copy number was calculated using 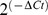 method (ΔCt = Difference in threshold cycle between test sample and endogenous control). The autosomal gene *RNase P* was used as an internal control.

### Statistical analysis

Statistical analysis was performed using Sigma Plot11.0. We tested the observed differences in the copy number of *BPY2*, *DAZ* and DYZ1 between the infertile males and NFMs using Fisher's exact test. A p-value 0.05 or less was considered statistically significant for the test.

## Supporting Information

Figure S1 **Alignment of** ***SRY*** **sequences from representative patients with the reference sequence from the GenBank (NM_003140.1).** The asterisk (*) below the sequence indicates the identical bases and the gaps indicate deletion of the nucleotide or putative mutations. The rectangular boxes are highlighting the insertions or deletions and the encircled red coloured bases are showing the nucleotide substitutions.
(DOCX)

Table S1 **Details of the STSs used for screening deletions of the** ***AZF*** **a, b and c regions.**
(DOCX)

Table S2 **Summary of representative candidate genes and STSs analyzed.**
(DOC)

Table S3 **Details of the primers used for end point PCR analyses.**
(DOC)

Table S4 **GeneBank accession numbers for cloned** ***SRY*** **sequences of the patients.**
(DOCX)

Table S5 **Clinical details of the patients' semen samples.**
(DOCX)

Table S6 **Details of the SNVs studied for** ***DAZ*** **,** ***GOLY-SNV-1*** **,** ***BPY2*** **and** ***TTTY4*** **genes.**
(DOCX)

Table S7 **List of the DYZ1 primers used for end point PCR, their corresponding amplicon sizes and reaction conditions.**
(DOCX)
