# Supplementary material for: Organizational and Functional Status of the Y-linked Genes and Loci in the Infertile Patients Having Normal Spermiogram
Source: PLoS One. 2012 Jul 23;7(7):e41488. doi: 10.1371/journal.pone.0041488 (PMC3402420; doi:10.1371/journal.pone.0041488)
Supplement: Figure S1 — Alignment of SRY sequences from representative patients with the reference sequence from the GenBank (NM_003140.1). The asterisk (*) below the sequence indicates the identical bases and the gaps indicate deletion of the nucleotide or putative mutations. The rectangular boxes are highlighting the insertions or deletions and the encircled red coloured bases are showing the nucleotide substitutions. (DOCX) [file pone.0041488.s001.docx]

Figure S1. Alignment of *SRY* sequences from representative patients with the reference (normal) sequence from the GenBank.

AS-9 GAATCTGGTAG--AAGTG-AGTTTTGGATAGTAAAA-TAAGTTTCGAA-CTCTGGCACCT 55

AS-11 GAATCTGGTAG--AAGTG-AGTTTTGGATAGTAAAA-TAAGTTTCGAA-CTCTGGCACCT 55

NORMAL GAATCTGGTAG--AAGTG-AGTTTTGGATAGTAAAA-TAAGTTTCGAA-CTCTGGCACCT 55

AS-15 GAATCTGGTAG--AAGTG-AGTTTTGGATAGTAAAA-TAAGTTTCGAA-CTCTGGCACCT 55

AS-29 GAATCTGGTAG--AAGTG-AGTTTTGGATAGTAAAA-TAAGTTTCGAA-CTCTGGCACCT 55

AS-28 GAATCTGGTAG--AAGTG-AGTTTTGGATAGTAAAAATAAGTTTCGA--CTCTGGCAC-T 54

AS-17 GAATCTGGTAG--AAGTG-AGTTTTGGATAGTAAAA-TAAGTTTCGAA-CTCTGGCACCT 55

AS-18 GAATCTGGTAAGAAAGTGGAGTTTTGGATAGTAAAATAAAGTTTCGAAACTCTGGCACCT 60

********** ***** ***************** ********* ********* *

AS-9 TTC--AATTTTGTCGCACTCTCCTTGTTTTTGACAATGCAATCATATGCTTCTGCTATGT 113

AS-11 TTC--AATTTTGTCGCACTCTCCTTGTTTTTGACAATGCAATCATATGCTTCTGCTATGT 113

NORMAL TTC--AATTTTGTCGCACTCTCCTTGTTTTTGACAATGCAATCATATGCTTCTGCTATGT 113

AS-15 TTC--AATTTTGT**T**GCACTCTCCTTGTTTTTGACAATGCAATCATATGCTTCTGCTATGT 113

AS-29 TTC--AATTTTGTCGCACTCTCCTTGTTTTTGACAATGCAATCATATGCTTCTGCTATGT 113

AS-28 TTC--AATTTTGTCGCACTCTCCTTGTTTTTGACAATGCAATCATATGCTTCTGCTATGT 112

AS-17 TTC--AATTTTGTCGCACTCTCCTTGTTTTTGACAATGCAATCATATGCTTCTGCTATGT 113

AS-18 TTTCAAATTTTGTCGCACTCTCCTTGTTTTTGACAATGCAATCATATGCTTCTGCTATGT 120

** ******** **********************************************

AS-9 TAA-GCGTATTCAA-CAGCGATGATTACAGTCCAGCTGTGCAAGAGAATATTCCCGCTCT 171

AS-11 TAA-GCGTATTCAA-CAGCGATGATTACAGTCCAGCTGTGCAAGAGAATATTCCCGCTCT 171

NORMAL TAA-GCGTATTCAA-CAGCGATGATTACAGTCCAGCTGTGCAAGAGAATATTCCCGCTCT 171

AS-15 TAA-GCGTATTCAA-CAGCGATGATTACAGTCCAGCTGTGCAAGAGAATATTCCCGCTCT 171

AS-29 TAA-GCGTATTCAA-CAGCGATGATTACAGTCCAGCTGTGCAAGAGAATATTCCCGCTCT 171

AS-28 TAA-GCGTATTCAAACAGCGATGATTACAGTCCAGCTGTGCAAGAGAATATTCCCGCTCT 171

AS-17 TAA-GCGTATTCAA-CAGCGATGATTACAGTCCAGCTGTGCAAGAGAATATTCCCGCTCT 171

AS-18 TAAAGCGTATTCAA-CAGCGATGATTACAGTCCAGCTGTGCAAGAGAATATTCCCGCTCT 179

*** ********** *********************************************

AS-9 CCGGAGAAGCTCTTCCTTCCTTTGCACTGAAAGCTGTAACTCTAAGTATCAGTGTGAAAC 231

AS-11 CCGGAGAAGCTCTTCCTTCCTTTGCACTGAAAGCTGTAACTCTAAGTATCAGTGTGAAAC 231

NORMAL CCGGAGAAGCTCTTCCTTCCTTTGCACTGAAAGCTGTAACTCTAAGTATCAGTGTGAAAC 231

AS-15 CCGGAGAAGCTCTTCCTTCCTTTGCACTGAAAGCTGTAACTCTAAGTATCAGTGTGAAAC 231

AS-29 CCGGAGAAGCTCTTCCTTCCTTTGCACTGAAAGCTGTAACTCTAAGTATCAGTGTGAAAC 231

AS-28 CCGGAGAAGCTCTTCCTTCCTT-GCACTGAAAGCTGTAACTCTAAGTATCAGTGTGAAAC 230

AS-17 CCGGAGAAGCTCTTCCTTCCTTTGCACTGAAAGCTGTAACTCTAAGTATCAGTGTGAAAC 231

AS-18 CCGGAGAAGCTCTTCCTTCCTTTGCACTGAAAGCTGTAACTCTAAGTATCAGTGTGAAAC 239

********************** *************************************

AS-9 GGGAGAAAACAGTAAAGGCAACGTCCAGGATAGAGTGAAGCGACCCATGAACGCATTCAT 291

AS-11 GGGAGAAA**G**CAGTAAAGGCAACGTCCAGGATAGAGTGAAGCGACCCATGAACGCATTCAT 291

NORMAL GGGAGAAAACAGTAAAGGCAACGTCCAGGATAGAGTGAAGCGACCCATGAACGCATTCAT 291

AS-15 GGGAGAAAACAGTAAAGGCAACGTCCAGGATAGAGTGAAGCGACCCATGAACGCATTCAT 291

AS-29 GGGAGAAAACAGTAAAGGCAACGTCCAGGATAGAGTGAAGCGACCCATGAACGCATTCAT 291

AS-28 GGGAGAAAACAGTAAAGGCAACGTCCAGGATAGAGTGAAGCGACCCATGAACGCATTCAT 290

AS-17 GGGAGAAAACAGTAAAGGCAACGTCCAGGATAGAGTGAAGCGACCCATGAACGCATTCAT 291

AS-18 GGGAGAAAACAGTAAAGGCAAC**A**TCCAGGATAGAGTGAAGCGACCCATGAACGCATTCAT 299

******** ************* *************************************

AS-9 CGTGTGGTCTCGCGATCAGAGGCGCAAGATGGCTCTAGAGAATCCC-AGAATGCGAAACT 350

AS-11 CGTGTGGTCTCGCGATCAGAGGCGCAAGATGGCTCTAGAGAATCCCCAGAATGCGAAACT 351

NORMAL CGTGTGGTCTCGCGATCAGAGGCGCAAGATGGCTCTAGAGAATCCC-AGAATGCGAAACT 350

AS-15 CGTGTGGTCTCGCGATCAGAGGCGCAAGATGGCTCTAGAGAATCCC-AGAATGCGAAACT 350

AS-29 CGTGTGGT**T**TCGCGATCAGAGGCGCAAGATGGCTCTAGAGAATCCC-AGAATGCGAAACT 350

AS-28 CGTGTGGTCTCGCGATCAGAGGCGCAAGATGGCTCTAGAGAATCCC-AGAATGCGAAACT 349

AS-17 CGTGTGGTCTCGCGATCAGAGGCGCAAGATGGCTCTAGAGAATCCC-AGAATGCGAAACT 350

AS-18 CGTGTGGTCTCGCGATCAGAGGCGCAAGATGGCTCTAGAGAATCCC-AGAATGCGAAACT 358

******** ************************************* *************

AS-9 CAGAGATCAGCAAGCAGCTGGG-ATACCAGTGGAAAATGCTTACTG-AAGCCGAAAAATG 408

AS-11 CAGAGATCAGCAAGCAGCTGGGGATACCAGTGGAAAATGCTTACTGGAAGCCGAAAAATG 411

NORMAL CAGAGATCAGCAAGCAGCTGGG-ATACCAGTGGAAAATGCTTACTG-AAGCCGAAAAATG 408

AS-15 CAGAGATCAGCAAGCAGCTGGG-ATACCAGTGGAAAATGCTTACTG-AAGCCGAAAAATG 408

AS-29 CAGAGATCAGCAAGCAGCTGGG-ATACCAGTGGAAAATGCTTACTG-AAGCCGAAAAATG 408

AS-28 CAGAGATCAGCAAGCAGCTGGG-ATACCAGTGGAAAATGCTTACTG-AAGCCGAAAAATG 407

AS-17 CAGAGATCAGCAAGCAGCTGGG-ATACCAGTGGAAAATGCTTACTG-AAGCCGAAAAATG 408

AS-18 CAGAGATCAGCAAGCAGCTGGG-ATACCAGTGGAAAATGCTTACTG-AAGCCGAAAAATG 416

********************** *********************** *************

AS-9 GCCATTCTTCCAGGAGGCACAGAAATTAC**C**GGCCCATGCACAGAGAGAAATACCCGAATT 468

AS-11 GCCAT-CTTCCAGGAGGCACAGAAATTACAGGCC-ATGCACAGAGAGAAATACCCGAATT 469

NORMAL GCCATTCTTCCAGGAGGCACAGAAATTACAGGCC-ATGCACAGAGAGAAATACCCGAATT 467

AS-15 GCCATTCTTCCAGGAGGCACAGAAATTACAGGCC-ATGCACAGAGAGAAATACCCGAATT 467

AS-29 GCCATTCTTCCAGGAGGCACAGAAATTACAGGCC-AT**C**CACAGAGAGAAATACCCGAATT 467

AS-28 GCCATTCTTCCAGGAGGCACAGAAATTACAGGCC-ATGCACAGAGAGAAATACCCGAATT 466

AS-17 GCCATTCTTCCAGGAGGCACAGAAATTACAGGCC-ATG**T**ACAGAGAGAAATACCCGAATT 467

AS-18 GCCATTCTTCCAGGAGGCACAGAAATTACAGGCC-ATGCACAGAGAGAAATACCCGAATT 475

***** *********************** **** ** *********************

AS-9 ATAAGTATCGACCTCGTCGGAAGGCGAAGATGCTGCCGAAGAATTGCAGTTTGCTTCCCG 528

AS-11 ATAAGTATCGACCTCGTCGGAAGGCGAAGATGCTGCCGAAGAATTGCAGTTTGCTTCCCG 529

NORMAL ATAAGTATCGACCTCGTCGGAAGGCGAAGATGCTGCCGAAGAATTGCAGTTTGCTTCCCG 527

AS-15 ATAAGTATCGACCTCGTCGGAAGGCGAAGATGCTGCCGAAGAATTGCAGTTTGCTTCCCG 527

AS-29 ATAAGTATCGACCTCGTCGGAAGGCGAAGATGCTGCCGAAGAATTGCAGTTTGCTTCCCG 527

AS-28 ATAAGTATCGACCTCGTCGGAAGGCGAAGATGCTGCCGAAGAATTGCAGTTTGCTTCCCG 526

AS-17 ATAAGTATCGACCTCGTCGGAAGGCGAAGATGCTGCCGAAGAATTGCAGTTTGCTTCCCG 527

AS-18 ATAAGTATCGACCTCGTCGGAAGGCGAAGATGCTGCCGAAGAATTGCAGTTTGCTTCCCG 535

************************************************************

AS-9 CAGATCCCGCTTCGGTACTCTGCAGCGAAG-GCAACTG-ACAACAGGTTGTACAGGGATG 586

AS-11 CAGATCCCGCTTCGGTACTCTGCAGCGAAGTGCAACTGGACAACAGGTTGTACAGGGATG 589

NORMAL CAGATCCCGCTTCGGTACTCTGCAGCGAAGTGCAACTGGACAACAGGTTGTACAGGGATG 587

AS-15 CAGATCCCGCTTCGGTACTCTGCAGCGAAGTGCAACTGGACAACAGGTTGTACAGGGATG 587

AS-29 CAGATCCCGCTTCGGTACTCTGCAGCGAAGTGCAACTGGACAACAGGTTGTACAGGGATG 587

AS-28 CAGATCCCGCTTCGGTACTCTGCAGCGAAGTGCAACTGGACAACAGGTTGTACAGGGATG 586

AS-17 CAGATCCCGCTTCGGTACTCTGCAGCGAAGTGCAACTGGACAACAGGTTGTACAGGGATG 587

AS-18 CAGATCCCGCTTCGGTACTCTGCAGCGAAGTGCAACTGGACAACAGGTTGTACAGGGATG 595

****************************** ******* *********************

AS-9 ACTGTACGAAAGCCACACACTCAAGAATGGAGCACCAGCTAGGCCACTTACCGCCCATCA 646

AS-11 ACTGTACGAAAGCCACACACTCAAGAATGGAGCACCAGCTAGGCCACTTACCGCCCATCA 649

NORMAL ACTGTACGAAAGCCACACACTCAAGAATGGAGCACCAGCTAGGCCACTTACCGCCCATCA 647

AS-15 ACTGTACGAAAGCCACACACTCAAGAATGGAGCACCAGCTAGGCCACTTACCGCCCATCA 647

AS-29 ACTGTACGAAAGCCACACACTCAAGAATGGAGCACCAGCTAGGCCACTTACCGCCCATCA 647

AS-28 ACTGTACGAAAGCCACACACTCAAGAATGGAGCACCAGCTAGGCCACTTACCGCCCATCA 646

AS-17 ACTGTACGAAAGCCACACACTCAAGAATGGAGCACCAGCTAGGCCACTTACCGCCCATCA 647

AS-18 ACTGTACGAAAGCCACACACTCAAGAATGGAGCACCAGCTAGGCCACTTACCGCCCATCA 655

************************************************************

AS-9 ACGCAGCCAGCTCACCGCAGCAACGGGACCGCTACAGCCACTGGACAAAGCTGTAGGACA 706

AS-11 ACGCAGCCAGCTCACCGCAGCAACGGGACCGCTACAGCCACTGGACAAAGCTGTAGGACA 709

NORMAL ACGCAGCCAGCTCACCGCAGCAACGGGACCGCTACAGCCACTGGACAAAGCTGTAGGACA 707

AS-15 ACGCAGCCAGCTCACCGCAGC**G**ACGGGACCGCTACAGCCACTGGACAAAGCTGTAGGACA 707

AS-29 ACGCAGCCAGCTCACCGCAGCAACGGGACCGCTACAGCCACTGGACAAAGCTGTAGGACA 707

AS-28 ACGCAGCCAGCTCACCGCAGCAACGGGACCGCTACAGCCACTGGACAAAGCTGTAGGACA 706

AS-17 ACGCAGCCAGCTCACCGCAGCAACGGGACCGCTACAGCCACTGGACAAAGCTGTAGGACA 707

AS-18 ACGCAGCCAGCTCACCGCAGCAACGGGACCGCTACAGCCACTGGACAAAGCTGTAGGACA 715

********************* **************************************

AS-9 ATCGGGTAACATTGGCTACAAAGACCTACCTAGATGCTCCTTTTTACGATAACTTACAGC 766

AS-11 ATCGGGTAACATTGGCTACAAAGACCTACCTAGATGCTCCTTTTTACGATAACTTACAGC 769

NORMAL ATCGGGTAACATTGGCTACAAAGACCTACCTAGATGCTCCTTTTTACGATAACTTACAGC 767

AS-15 ATCGGGTAACATTGGCTACAAAGACCTACCTAGATGCTCCTTTTTACGATAACTTACAGC 767

AS-29 ATCGGGTAACATTGGCTACAAAGACCTACCTAGATGCTCCTTTTTACGATAACTTACAGC 767

AS-28 ATCGGGTAACATTGGCTACAAAGACCTACCTAGATGCTCCTTTTTACGATAACTTACAGC 766

AS-17 ATCGGGTAACATTGGCTACAAAGACCTACCTAGATGCTCCTTTTTACGATAACTTACAGC 767

AS-18 ATCGGGTAACATTGGCTACAAAGACCTACCTAGATGCTCCTTTTTACGATAACTTACAGC 775

************************************************************

AS-9 CCTCACTTTCTTATGTTTAGTTTCAATATTGTTTTCTTTTCTCTGGCTAATAAAGGC 823

AS-11 CCTCACTTTCTTATGTTTAGTTTCAATATTGTTTTCTTTTCTCTGGCTAATAAAGGC 826

NORMAL CCTCACTTTCTTATGTTTAGTTTCAATATTGTTTTCTTTTCTCTGGCTAATAAAGGC 824

AS-15 CCTCACTTTCTTATGTTTAGTTTCAATATTGTTTTCTTTTCTCTGGCTAATAAAGGC 824

AS-29 CCTCACTTTCTTATGTTTAGTTTCAATATTGTTTTCTTTTCTCTGGCTAATAAAGGC 824

AS-28 CCTCACTTTCTTATGTTTAGTTTCAATATTGTTTTCTTTTCTCTGGCTAATAAAGGC 823

AS-17 CCTCACTTTCTTATGTTTAGTTTCAATATTGTTTTCTTTTCTCTGGCTAATAAAGGC 824

AS-18 CCTCACTTTCTTATGTTTAGTTTCAATATTGTTTTCTTTTCTCTGGCTAATAAAGGC 833

*********************************************************
