# Supplementary material for: Organizational and Functional Status of the Y-linked Genes and Loci in the Infertile Patients Having Normal Spermiogram
Source: PLoS One. 2012 Jul 23;7(7):e41488. doi: 10.1371/journal.pone.0041488 (PMC3402420; doi:10.1371/journal.pone.0041488)
Supplement: Table S1 — Details of the STSs used for screening deletions of the AZF a, b and c regions. (DOCX) [file pone.0041488.s002.docx]

**Table S1. Details of the STSs used for screening deletions of the *AZF*a, b and c regions**

| **S. No.** | ***AZF* region** | **STSs used** |
| --- | --- | --- |
| **1** | *AZF*a | sY95, sY746, sY1064, sY1065, sY1066, sY1179, sY1180, sY1181, sY1182, sY1183, sY1184, sY1185, sY1186, sY1231 (*UTY* exon 8), sY1234 (*DBY* exon 9), sY1251 (Boundary between Centromere and Yq), sY1316 (*USP9Y* exon 26), sY1317 (*USP9Y* exon3) |
| **2** | *AZF*b | sY117, sY125, sY127, sY129, sY113, sY131, sY627 (*RBMY* exon 12) |
| **3** | *AZF*c | sY121 (Immediately distal to palindrome 4), sY142 (proximal to *AZF*c), sY254 (*DAZ* exon 3), sY255, sY278, sY277, sY1054 (Blue/yellow boundaries in *AZF*c), sY1125 (Blue/gray boundaries in *AZF*c), sY1161 (*PRY* intron 2), sY1190 (yellow amplicons in *AZF*c), sY1191 (Unique sequence u3 in *AZF*c), sY1197(internal boundary of palindrome P3), sY1201 (Distal boundary of gray amplicon), sY1206 (yellow/green boundaries in *AZF*c), sY1246 (proximal portion of distal Yq heterochromatin), sY1258(Boundary between unique sequence u1 and blue amplicon b1 in *AZF*c) sY1263 (*CDY1* exon1/intron1), sY1291 (Red/gray boundary in *AZF*c), sY1322, sY1682 (*RSP4Y2* exon1) |
| **4** | Gene specific STSs | *DBY1*, *DBY2* (For *DBY* gene), F19/E355 (For *RBMY*), sY1035 (*BPY2* intron 5), sY1235 (*XKRY* exon 1), sY1233 (E1F1AY exon 1), sY1237 (*HSFY* exon 2), sY1260 (*CDY2*), sY1318 (*DAZ* exon 11), sY276(*AMELY* exon4/intron 4), sY1238, sY1240, sY1250 (proximal boundary of *TSPY*) and sY1319, *ZFY*, sY14 (*SRY*), RRM3, Y-DAZ3, sY152(*DAZ*) |
