# Supplementary material for: Organizational and Functional Status of the Y-linked Genes and Loci in the Infertile Patients Having Normal Spermiogram
Source: PLoS One. 2012 Jul 23;7(7):e41488. doi: 10.1371/journal.pone.0041488 (PMC3402420; doi:10.1371/journal.pone.0041488)
Supplement: Table S2 — Summary of representative candidate genes and STSs analyzed. (DOC) [file pone.0041488.s003.doc]

**Table S2. Summary of representative candidate genes and STSs analyzed**

| **S. No.** | ***SRY*1** | ***SRY* 2** | ***DBY1*** | ***CDY1*(1)** | ***CDY2*(1)** | ***XKRY*** | ***CDY1*(2)** | ***CDY2*(2)** | ***VCY*(1)** | ***VCY*(2)** | ***TTTY17*(1)** | ***TTTY17*(2)** | ***TTTY3*(1)** | ***TTTY3*(2)** | ***RPS4Y*** | **sY1231** | **sY1251** | **Y-DAZ3** | **sY152** |
| --- | --- | --- | --- | --- | --- | --- | --- | --- | --- | --- | --- | --- | --- | --- | --- | --- | --- | --- | --- |
| **AS-1** | **+** | **+** | **+** | **+** | **+** | **+** | **+** | **+** | **+** | **+** | **+** | **+** | **+** | **+** | **+** | **+** | **+** | **+** | **+** |
| **AS-2** | **+** | **+** | **+** | **+** | **-** | **+** | **+** | **-** | **+** | **+** | **+** | **+** | **+** | **+** | **+** | **+** | **+** | **+** | **+** |
| **AS-3** | **+** | **+** | **-** | **+** | **-** | **+** | **+** | **-** | **+** | **+** | **+** | **+** | **+** | **+** | **+** | **+** | **+** | **-** | **+** |
| **AS-4** | **+** | **+** | **-** | **+** | **+** | **+** | **+** | **+** | **+** | **+** | **+** | **+** | **+** | **+** | **+** | **+** | **+** | **-** | **+** |
| **AS-5** | **+** | **+** | **-** | **+** | **+** | **+** | **+** | **+** | **+** | **+** | **+** | **+** | **+** | **+** | **+** | **+** | **+** | **-** | **+** |
| **AS-6** | **+** | **+** | **-** | **+** | **-** | **+** | **+** | **-** | **+** | **+** | **+** | **+** | **+** | **+** | **+** | **+** | **+** | **-** | **+** |
| **AS-7** | **+** | **+** | **-** | **+** | **-** | **+** | **+** | **-** | **+** | **+** | **+** | **+** | **+** | **+** | **+** | **+** | **+** | **+** | **+** |
| **AS-8** | **+** | **+** | **-** | **+** | **+** | **+** | **+** | **+** | **+** | **+** | **+** | **+** | **+** | **+** | **+** | **+** | **+** | **+** | **+** |
| **AS-9** | **+** | **+** | **-** | **+** | **-** | **-** | **+** | **-** | **-** | **-** | **-** | **-** | **+** | **-** | **-** | **-** | **+** | **+** | **-** |
| **AS-10** | **+** | **+** | **-** | **+** | **+** | **+** | **+** | **+** | **+** | **+** | **+** | **+** | **+** | **+** | **+** | **+** | **+** | **-** | **+** |
| **AS-11** | **+** | **+** | **-** | **+** | **+** | **+** | **+** | **+** | **+** | **+** | **+** | **+** | **+** | **+** | **+** | **+** | **+** | **-** | **+** |
| **AS-12** | **+** | **+** | **-** | **+** | **-** | **+** | **+** | **-** | **+** | **+** | **+** | **+** | **+** | **+** | **+** | **+** | **+** | **-** | **+** |
| **AS-13** | **+** | **+** | **+** | **+** | **+** | **+** | **+** | **+** | **+** | **+** | **+** | **+** | **+** | **+** | **+** | **+** | **+** | **+** | **+** |
| **AS-14** | **+** | **+** | **-** | **+** | **+** | **+** | **+** | **+** | **+** | **+** | **+** | **+** | **+** | **+** | **+** | **+** | **+** | **-** | **+** |
| **AS-15** | **+** | **+** | **-** | **+** | **+** | **+** | **+** | **+** | **+** | **+** | **+** | **+** | **+** | + | **+** | **+** | **+** | **+** | **+** |
| **AS-16** | **+** | **+** | **-** | **+** | **-** | **-** | **+** | **-** | **-** | **-** | **-** | **-** | **+** | **+** | **-** | **+** | **+** | **+** | **-** |
| **AS-17** | **+** | **+** | **-** | **+** | **+** | **+** | ***+*** | ***+*** | **+** | **+** | **+** | **+** | **+** | **+** | **+** | **+** | **+** | **+** | **+** |
| **AS-18** | **+** | **+** | **-** | **+** | **+** | **+** | **+** | **+** | **+** | **+** | **+** | **+** | **+** | **+** | **+** | **+** | **+** | **+** | **+** |
| **AS-19** | **+** | **+** | **-** | **+** | **+** | **+** | **+** | **+** | **+** | **+** | **+** | **+** | **+** | **+** | **+** | **+** | **+** | **+** | **+** |
| **AS-20** | **+** | **+** | **-** | **+** | **+** | **+** | **+** | **+** | **+** | **+** | **+** | **+** | **+** | **+** | **+** | **+** | **+** | **+** | **+** |
| **AS-21** | **+** | **+** | **-** | **+** | **+** | **+** | **+** | **+** | **+** | **+** | **+** | **+** | **+** | **+** | **+** | **+** | **+** | **+** | **+** |
| **AS-22** | **+** | **+** | **-** | **+** | **-** | **+** | **+** | **-** | **+** | **+** | **+** | **+** | **+** | **+** | **+** | **+** | **+** | **-** | **+** |
| **AS-23** | **+** | **+** | **+** | **+** | **+** | **+** | **+** | **+** | **+** | **+** | **+** | **+** | **+** | **+** | **+** | **+** | **+** | **+** | **+** |
| **AS-24** | **+** | **+** | **+** | **+** | **+** | **+** | **+** | **+** | **+** | **+** | **+** | **+** | **+** | **+** | **+** | **+** | **+** | **+** | **+** |
| **AS-25** | **+** | **+** | **-** | **+** | **+** | **+** | **+** | **+** | **+** | **+** | **+** | **+** | **+** | **+** | **+** | **+** | **+** | **+** | **+** |
| **AS-26** | **+** | **+** | **+** | **+** | **-** | **+** | **+** | **-** | **+** | **+** | **+** | **+** | **+** | **+** | **+** | **+** | **+** | **+** | **+** |
| **AS-27** | **+** | **+** | **+** | **+** | **+** | **+** | **+** | **+** | **+** | **+** | **+** | **+** | **+** | **+** | **+** | **+** | **+** | **+** | **+** |
| **AS-28** | **+** | **+** | **+** | **+** | **+** | **+** | **+** | **+** | **+** | **+** | **+** | **+** | **+** | **+** | **+** | **+** | **+** | **+** | **+** |
| **AS-29** | **+** | **+** | **+** | **+** | - | **+** | **+** | **-** | **+** | **+** | **+** | **+** | **+** | **+** | **+** | **+** | **+** | **-** | **+** |
| **AS-30** | **+** | **+** | **+** | **+** | - | **+** | **+** | **-** | **+** | **+** | **+** | **+** | **+** | **+** | **+** | **+** | **+** | **+** | **+** |
| **AS-31** | **+** | **+** | **+** | **+** | - | **+** | **+** | **-** | **+** | **+** | **+** | **+** | **+** | **+** | **+** | **+** | **+** | **+** | **+** |
