# Supplementary material for: Organizational and Functional Status of the Y-linked Genes and Loci in the Infertile Patients Having Normal Spermiogram
Source: PLoS One. 2012 Jul 23;7(7):e41488. doi: 10.1371/journal.pone.0041488 (PMC3402420; doi:10.1371/journal.pone.0041488)
Supplement: Table S3 — Details of the primers used for end point PCR analyses. (DOC) [file pone.0041488.s004.doc]

**Table S3. Details of the primers used for end point PCR**

| **Primer** | **Sequence** |
| --- | --- |
| ***DYZ1*-1** | TTTCCTTTCGCTTGCATTCCAT |
| ***DYZ1*-2** | TTTTGAGTCCGTTCCATAACAC |
| ***DYZ1*-3** | GAGTCCATTCACTTCCAGAACA |
| ***DYZ1*-4** | CCATGCCATTTTATTGCGTTGC |
| ***DYZ1*-5** | GACTGGAAAGGCTGGGTGTCGA |
| ***DYZ1*-6** | TGAAATGGACTGGAAAGGAATG |
| ***DYZ1*-7** | TGGAATGGACTGCAATAGAAAG |
| ***DYZ1*-8** | TGGAATGGACTCGAACAGAGTG |
| ***SRY*1** | F- GAATCTGGTAGAAGTGAGTTTTGGA  R- GCCTTTATTAGCCAGAGAAAAGAAA |
| ***SRY*2** | F- CTTCTGCTATGTTAAGCGTATTCAA  R- CAGCTTTGTCCAGTGGCTGTA |
| **sY14** | F- GAATATTCCCGCTCTCCGGA  R- GCTGGTGCTCCATTCTTGAG |
| **β-actin** | F- AGATGACCCAGATCATGTTTGAGA  R-CTAAGTCATAGTCCGCCTAGAAGC |
| ***USP9Y1*** | F- AGGGAATTAGCGAGCTTGAAAC  R- AAATATCCTGCCCCAGCT TAG |
| ***USP9Y2*** | F- TGCAAGATGTTTTGTCCTTGAA  R- AATCATTCAGGACATGTTTCACAA |
| ***USP9Y3*** | F- GAGCCCATCTTTGTCAGTTTAC  R- CTGCCAATTTTCCACATCAACCC |
| ***DBY*** | F- ATCGACAAAGTAGTGGTTCC  R- AGATTCAGTTGCCCCACCAG |
| ***DBY1*** | F- TATTGGCAATCGTGAAAGAC  R- TGCCGGTTGCCTCTACTGGT |
| ***DBY2*** | F- ATCGACAAAGTAGTGGTTCC  R- AGATTCAGTTGCCCCACCAG |
| ***CDY1*(1)** | F- GGCGAAAGCTGACAGCAA  R- GGGTGAAAGTTCCAGTCAA |
| ***CDY1*(2)** | F- AAAGCTTTCTGTACTACACCAGAGGGTTG  R- AAGAAGTTTCTGCCTTTAATAATGTGTCCA |
| ***CDY2*(1)** | F- GACCACAAGAAAACTGTGAG  R- GATCTGCTGCAATAGGGTC |
| ***CDY2*(2)** | F- AAAGCTTTCTGTACTACACCAGAGGGTTG  R- AGCAGACAGACTGACAATTAAAACTATCA |
| ***TTTY17*(1)** | F- TCTGACCATGACGTGTTTCTTG  R- ACACTGCATGCTTTTTCCAAG |
| ***TTTY17* (2)** | F- CAGTGGAGGTTCTTCTTCCTG  R- TCTTTTCTGGTCTCCATTCCA |
| ***TTTY3*(1)** | F- TCTGGCCTAGACTTAGATTTTGGAA  R- GACCAGAAATGAAGACCAGTTGAA |
| ***TTTY3*(2)** | F- GCCCTAGAGAAGCTCATTGCAT  R- TCAGTTACCAGTGTCCCAGCAT |
| ***RPS4Y*** | F- AGTTGTGCAAAGTGAGGAAGATTA  R- CAGCCTCTTATCTCTCTCTTCAGC |
| ***VCY*(1)** | F- TAGTGGAGTGTTGACCAATCACAG  R- ACACACCACCTCTTCCTTCCTC |
| ***VCY*(2)** | F- AAGCCAGTGCTTTAAACCATGAGAA  R-TCAGAGAACACACAGAGTCACTCAGATCA |
