# Supplementary material for: Organizational and Functional Status of the Y-linked Genes and Loci in the Infertile Patients Having Normal Spermiogram
Source: PLoS One. 2012 Jul 23;7(7):e41488. doi: 10.1371/journal.pone.0041488 (PMC3402420; doi:10.1371/journal.pone.0041488)
Supplement: Table S4 — GeneBank accession numbers for cloned SRY sequences of the patients. (DOCX) [file pone.0041488.s005.docx]

**Table S4. GeneBank accession numbers for cloned *SRY* sequences of the patients**

| **S. No.** | **Patients’ ID** | **Accession number** |
| --- | --- | --- |
| **1** | AS-1 | JQ811904 |
| **2** | AS-2 | JQ811905 |
| **3** | AS-3 | JQ811906 |
| **4** | AS-4 | JQ811907 |
| **5** | AS-5 | JQ811908 |
| **6** | AS-6 | JQ811909 |
| **7** | AS-7 | JQ811910 |
| **8** | AS-8 | JQ811911 |
| **9** | AS-9 | JQ811912 |
| **10** | AS-10 | JQ811913 |
| **11** | AS-11 | JQ811914 |
| **12** | AS-12 | JQ811915 |
| **13** | AS-13 | JQ811916 |
| **14** | AS-14 | JQ811917 |
| **15** | AS-15 | JQ811918 |
| **16** | AS-16 | JQ811919 |
| **17** | AS-17 | JQ811920 |
| **18** | AS-18 | JQ811921 |
| **19** | AS-19 | JQ811922 |
| **20** | AS-20 | JQ811923 |
| **21** | AS-21 | JQ811924 |
| **22** | AS-22 | JQ811925 |
| **23** | AS-23 | JQ811926 |
| **24** | AS-24 | JQ811927 |
| **25** | AS-25 | JQ811928 |
| **26** | AS-26 | JQ811929 |
| **27** | AS-27 | JQ811930 |
| **28** | AS-28 | JQ811931 |
| **29** | AS-29 | JQ811932 |
| **30** | AS-30 | JQ811933 |
| **31** | AS-31 | JQ811934 |
