# Supplementary material for: Organizational and Functional Status of the Y-linked Genes and Loci in the Infertile Patients Having Normal Spermiogram
Source: PLoS One. 2012 Jul 23;7(7):e41488. doi: 10.1371/journal.pone.0041488 (PMC3402420; doi:10.1371/journal.pone.0041488)
Supplement: Table S5 — Clinical details of the patients' semen samples. (DOCX) [file pone.0041488.s006.docx]

**Table S5. Clinical details of the patients’ semen samples**

| **Patients’ ID** | **Age** | **Sperm parameters** | | | |
| --- | --- | --- | --- | --- | --- |
|  |  | **Semen Volume (ml)** | **Sperm count (millions/ml)** | **Sperm motility (%)** | **Sperm Viability (%)** |
| **AS-1** | 28 | 3.5 | 100 | 50 | 60 |
| **AS-2** | 25 | 4 | 140 | 50 | 60 |
| **AS-3** | 31 | 1.5 | 120 | 50 | 60 |
| **AS-4** | 28 | 2 | 40 | 60 | 60 |
| **AS-5** | 29 | 1.5 | 180 | 40 | 60 |
| **AS-6** | 36 | 3.8 | 140 | 80 | 75 |
| **AS-7** | 37 | 3.6 | 100 | 30 | 60 |
| **AS-8** | 40 | 4 | 120 | 80 | 75 |
| **AS-9** | 37 | 3 | 150 | 50 | 55 |
| **AS-10** | 29 | 2.8 | 60 | 60 | 55 |
| **AS-11** | 40 | 2 | 60 | 80 | 70 |
| **AS-12** | 36 | 4 | 120 | 60 | 70 |
| **AS-13** | 36 | 4 | 40 | 55 | 50 |
| **AS-14** | 31 | 2 | 40 | 55 | 55 |
| **AS-15** | 34 | 1.5 | 70 | 80 | 60 |
| **AS-16** | 29 | 3.6 | 180 | 80 | 70 |
| **AS-17** | 47 | 4.5 | 180 | 55 | 50 |
| **AS-18** | 32 | 1.5 | 70 | 55 | 60 |
| **AS-19** | 32 | 2 | 180 | 80 | 70 |
| **AS-20** | 28 | 1.5 | 80 | 70 | 70 |
| **AS-21** | 27 | 2 | 80 | 60 | 60 |
| **AS-22** | 33 | 1.5 | 40 | 50 | 55 |
| **AS-23** | 34 | 2.6 | 160 | 80 | 75 |
| **AS-24** | 33 | 1.5 | 100 | 80 | 70 |
| **AS-25** | 36 | 1.8 | 180 | 80 | 70 |
| **AS-26** | 30 | 2 | 60 | 60 | 60 |
| **AS-27** | 24 | 2.5 | 70 | 55 | 60 |
| **AS-28** | 36 | 2 | 65 | 60 | 50 |
| **AS-29** | 24 | 2.1 | 60 | 70 | 55 |
| **AS-30** | 25 | 2 | 55 | 50 | 60 |
| **AS-31** | 39 | 2 | 90 | 50 | 60 |
