# Supplementary material for: Organizational and Functional Status of the Y-linked Genes and Loci in the Infertile Patients Having Normal Spermiogram
Source: PLoS One. 2012 Jul 23;7(7):e41488. doi: 10.1371/journal.pone.0041488 (PMC3402420; doi:10.1371/journal.pone.0041488)
Supplement: Table S6 — Details of the SNVs studied for DAZ , GOLY-SNV-1 , BPY2 and TTTY4 genes. (DOCX) [file pone.0041488.s007.docx]

**Table S6. Details of the SNVs studied for *DAZ*, *GOLY*-SNV_1, *BPY2* and *TTTY4* genes**

| **Target** | **SNV** | **Oligos used** | **Accession no.** | **Product size** | **Enzyme for digestion** | **Restriction site** | **Fragments** | **Alleles** | **Present in copies** |
| --- | --- | --- | --- | --- | --- | --- | --- | --- | --- |
|  | *DAZ*-SNV_I | SA 770 CACAGGCACTCAGTAACTATCTC  SA771 CAGTGTTTCACCCACCACTTCTGGGT | [*G73167*](http://www.ncbi.nlm.nih.gov/nucleotide/G73167) | 709 | *Fsp1* | TGC/GCA | 709  398+311 | A  B | 1,2,3 4 |
|  | *DAZ*-SNV_II | SA 446 GACATCCACGTCATTAACAAACG  SA 447 GGAAGCTGCTTTGGTAGATAC | [*G73166*](http://www.ncbi.nlm.nih.gov/nucleotide/G73166) | 182 | *Mbo1* | /GATC | 182  122+60 | A  B | 1 2,3,4 |
|  | DAZ-SNV_III (sY586) | SA 772 GTGTGGCACATATGCCTATAAA  SA 773 TTGGTACATCCAGATGCAGAT | [*G63907*](http://www.ncbi.nlm.nih.gov/nucleotide/G63907) | 301 | *Taq1* | T/CGA | 301  184+117 | A  B | 2 1,3,4 |
| ***DAZ* gene** | *DAZ*-SNV_IV | SA 774 CTTCCTCATCTTTCTTGACTT  SA 775 TTATTTATTCCTCAAAAAGGTG | [*G73168*](http://www.ncbi.nlm.nih.gov/nucleotide/G73168) | 630 | *AluI* | AG/CT | 630  398+262 | A  B | 2 1,3,4 |
|  | DAZ-SNV_V (sY587) | SA 444 TGGTTAATAAAGGGAAGGTGTTTT  SA 445 TCTCCAGGACAGGAAAATCC | [*G63908*](http://www.ncbi.nlm.nih.gov/nucleotide/G63908) | 244 | *DraI* | TTT/AAA | 195+49  122+73+49 | A  B | 3,4 1,2 |
|  | *DAZ*-SNV_VI | SA 776 GGGCCTAGTCTCTAGATCATT  SA 777 GCTAGAACCAAATATTCTGGAT | [*G73169*](http://www.ncbi.nlm.nih.gov/nucleotide/G73169) | 431 | *AflIII* | A/CRYGT | 431  248+183 | A  B | 1,2,3 4 |
|  | DAZ-SNV_VII (sY581) | SA 442 CACTGCCCTAATCCTAGCACA  SA 443 TCTTCTGGACATCCACGTCA | [*G63906*](http://www.ncbi.nlm.nih.gov/nucleotide/G63906) | 252 | *Sau3AI* | /GATC | 189+63  130+63+59 | A  B | 1,4 2,3 |
| ***GOLY1*** | *GOLY*-SNV_1 | SA768 TTGGCCTGTTGCTTCTAGGGTT  SA769 ACAGGGAGGGTGCTGTCACA | [*BV012733*](http://www.ncbi.nlm.nih.gov/nucleotide/BV012733) | 531 | *HhaI* | GCG/C | 531  289+282 | A  B | 1 Copy  1 Copy |
| ***BPY2*** | *BPY2* | SA766 AAGCCCATTGCTGAGATACTG  SA767 TTGTGATTCTGACCCAACGA | [*BV012732*](http://www.ncbi.nlm.nih.gov/nucleotide/BV012732) | 470 | *EcoRV* | GAT/ATC | 470  289+181 | A  B | 2 Copy  1 Copy |
| ***TTTY4*** | *TTTY4* | SA764 TGCAGACAGCACTGTGGCTT  SA765 GTATATGGCATAATTTCACCTG | [*BV012731*](http://www.ncbi.nlm.nih.gov/nucleotide/BV012731) | 541 | *HaeIII* | GG/CC | 541  323+218 | A  B | 1 Copy  2 Copy |
