# Supplementary material for: Organizational and Functional Status of the Y-linked Genes and Loci in the Infertile Patients Having Normal Spermiogram
Source: PLoS One. 2012 Jul 23;7(7):e41488. doi: 10.1371/journal.pone.0041488 (PMC3402420; doi:10.1371/journal.pone.0041488)
Supplement: Table S7 — List of the DYZ1 primers used for end point PCR, their corresponding amplicon sizes and reaction conditions. (DOCX) [file pone.0041488.s008.docx]

**Table S7. List of the *DYZ*1 primers used for end point PCR,**

**their corresponding amplicon sizes and reaction conditions**

| **Primer combination**  **(PC)** | **Primers** | **Amplicon Size (bp)** | **PCR conditions** |
| --- | --- | --- | --- |
| **1** | *DYZ*1(1 & 5) | 3378 | 64-1.5’, 72-3.5’ |
| **2** | *DYZ*1( 1 & 6) | 266 | 60-1’, 72-1’ |
| **3** | *DYZ*1(1 & 7 ) | 1564 | 60-1’, 72-1’ |
| **4** | *DYZ*1(1 & 8 ) | 2095 | 60-1’, 72-2’ |
| **5** | *DYZ*1(2 & 5 ) | 2056 | 62-1.5’, 72-3’ |
| **6** | *DYZ*1(2 & 7) | 242 | 61-1’, 72-1’ |
| **7** | *DYZ*1(2 & 8) | 773 | 59-1’, 72-1’ |
| **8** | *DYZ*1(3 & 5) | 275 | 63-1’, 72-1’ |
| **9** | *DYZ*1(4 & 5) | 1612 | 60-1’, 72-2’ |
| **10** | *DYZ*1(4 & 8) | 329 | 60-1’, 72-1’ |
